# Possibility of Avoiding Anesthesia in the Reduction of Greenstick and Angulated Forearm and Distal-End Radius Fractures in Children: A Comparative Study

**DOI:** 10.7759/cureus.38966

**Published:** 2023-05-13

**Authors:** Sanjay Rai, Mahendrakumar C Bendale, Mohit Hanwate, Deepak Reddy, Arjun Gandotra

**Affiliations:** 1 Orthopaedics, Military Hospital, Ambala, IND; 2 Orthopaedics, SMBT (Smt Mathurabai Bhausaheb Thorat) Medical College, Nashik, IND; 3 Orthopaedics, SMBT (Smt Mathurabai Bhausaheb Thorat) Institute of Medical Sciences and Research Centre, Nashik, IND; 4 Radiology, Military Hospital, Ambala, IND; 5 Orthopaedics, Dr C Lal Hospital, Ambala, IND

**Keywords:** closed reduction, anaesthesia, fracture angulation, children, greenstick fracture

## Abstract

Introduction

Greenstick and angulated forearm bone fractures are the most common fractures in children and invariably require closed reduction under anesthesia. However, pediatric anesthesia is somewhat risky and not always available in developing countries like India. Therefore, this study aimed to evaluate the standard (quality) of closed reduction without anesthesia in children and to determine satisfaction among parents.

Materials and methods

The present study included 163 children with closed angulated fractures of the distal radius and fracture shafts of both forearm bones, who were treated by closed reduction. One hundred and thirteen were treated without any anesthesia (study group) on an outpatient department (OPD) basis, whereas 50 children of similar age and fracture type underwent reduction with anesthesia (control group). After reduction by both methods check X-ray was done to evaluate the quality of the reduction.

Results

The average age of the 113 children in the present study was 9.5 years (range: 3.5-16.2 years), of which 82 children had radius or ulna fractures, and 31 had isolated distal radius fractures. In 96.8% of children, ≤10° of residual angulation was achieved. Furthermore, 11 children (12.4%) used paracetamol or ibuprofen for pain control in the study group. Moreover, 97.3% of parents stated that they would like their children to be treated without anesthesia if any fracture occurred again.

Conclusions

Closed reduction of greenstick angulated forearm and distal-end radius fracture in children in the OPD without anesthesia achieved satisfactory reduction and high parent satisfaction while reducing the risks of pediatric anesthesia and its associated complications.

## Introduction

Forearm bone and wrist fractures are estimated to account for 25%-45% of all pediatric fractures and are the most common fractures in children [1-4]. However, some estimate this value to be as high as 30%-50% [5-7], and these fractures have shown an increasing trend in recent years [8,9]. The incidence of forearm bone fractures in children aged up to 16 years is 0.7 in 1000 [10,11]. Sinikumpu et al. recorded that 18% of children experience a fracture by the age of nine, with children between the ages of five and 14 having the highest fracture incidence [12].

The majority of forearm and wrist bone fractures can be successfully treated using conservative means with closed reduction and plaster application. These children invariably require anesthesia, and the options vary from Bier blocks, hematoma blocks, nerve blocks, intravenous (IV) sedation, and general anesthesia. Approximately 78%-80% of surgeons use some kind of anesthesia during closed reduction [13-16]. Nemeth recommends the reduction of all pediatric fractures in the operating room whenever possible, as this approach reduces patient distress, relieves anxiety, manages pain, and minimizes distractions for the treating surgeon [17]. However, many reduce fractures under awake sedation in the emergency room with good outcomes [18-20]. Closed reduction under anesthesia offers painless and better muscle relaxation, which makes the reduction maneuver easier to perform. However, no anesthesia is free from complications, and there have been no studies to support these notions to date.

If a reduction is performed under general anesthesia, many side effects and complications can occur ranging from nausea and vomiting, anaphylaxis, arrhythmia, seizure, hypotension, malignant hyperthermia, aspiration pneumonitis, respiratory depression, and sometimes even death [21-24]. Schneuer et al. reported that children who were given general anesthesia before age four had poorer development during their school entry and school performance [24]. A meta-analysis by Di Maggio et al. showed that general anesthesia can cause neurodevelopmental impairment in children [25].

Although regional anesthetics typically have fewer complications compared to general anesthesia, some children may require sedation or general anesthesia if regional anesthesia produces either inadequate pain relief or analgesia before closed reduction [26-28]. In developing countries and others, treatment costs increase if any type of anesthesia is used. Furthermore, if general anesthesia is given, 4-6 hours of observation are required, and, especially, in India, where very few people are medically insured, it is a costly affair. In India, parents invariably prefer closed reduction without any anesthesia on an outpatient department (OPD) basis so that they can leave the hospital as soon as possible. Although it is highly likely that many surgeons in India have been doing closed reductions of angulated and greenstick pediatric forearm fractures without any anesthesia, the outcomes have not been published in the literature.

In light of the above discussion, this study aimed to evaluate the standard of closed reduction in children with forearm and wrist fractures performed without any use of anesthesia and to determine parents’ satisfaction with these methods. We hypothesized that closed reduction of these fractures without anesthesia would produce satisfactory alignment and high parent satisfaction.

## Materials and methods

**Figure 1:**
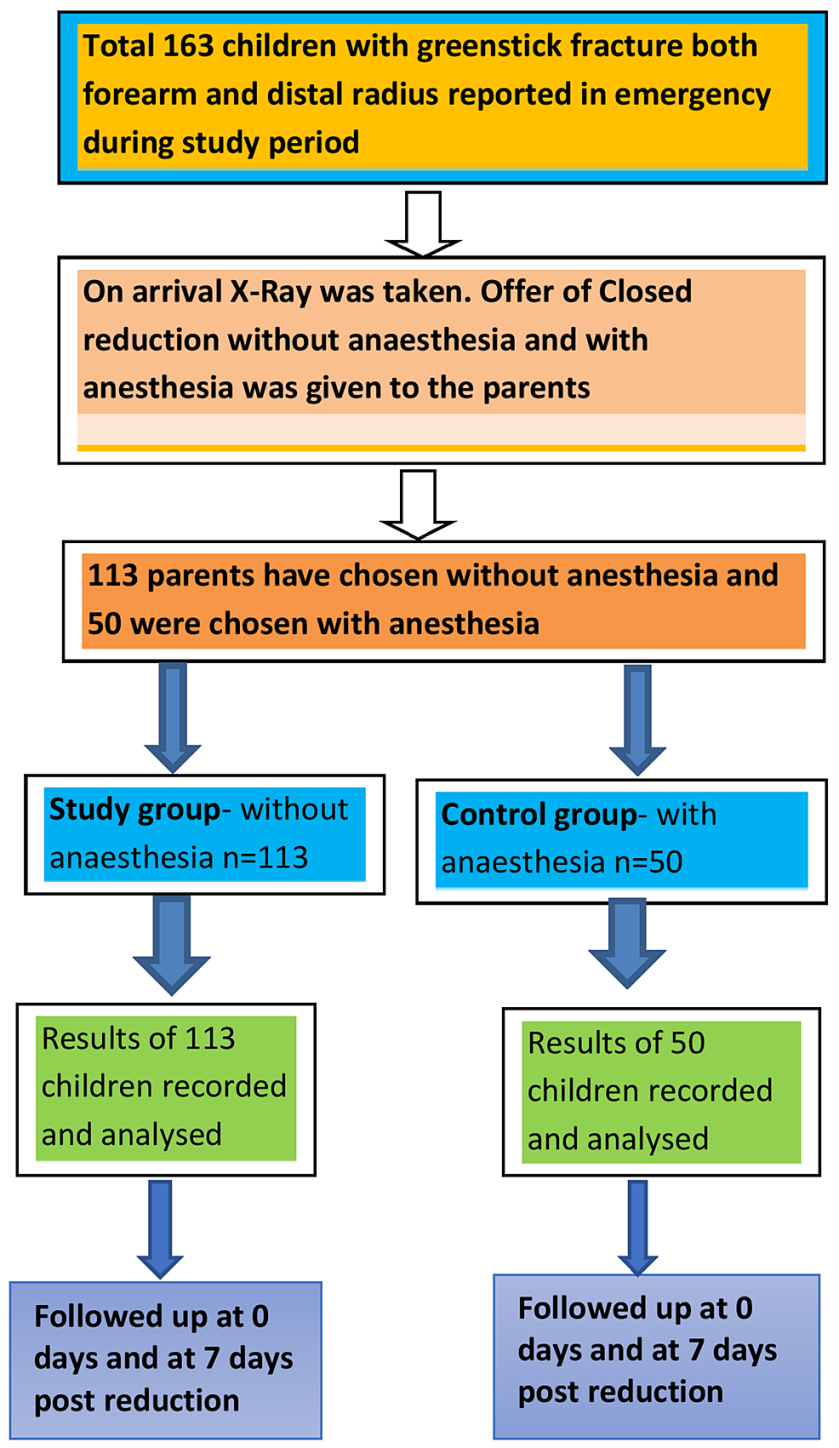
Study design

This was a prospective study of 163 children with greenstick distal radius fractures and angulated fractures of the shaft radius or ulna treated by closed reduction and casting. The children’s parents were asked to opt for having or not having anesthesia in closed reduction. The entire procedure had been fully explained to the parents and a written informed consent was taken.* *One hundred and thirteen parents chose closed reduction without anesthesia for their children (the study group), and 50 parents chose closed reduction with anesthesia (the control group), as shown in Figure 1.

The 50 children in the control group were of the same age and had the same type of fracture, where the closed reduction was done using some kind of anesthesia. Approval was obtained from the institutional review board of Military Hospital Ambala, Haryana, India before the study was performed (Approval No. MAH/Ortho/July 2017). The study was conducted between September 2017 and July 2022. Informed consent was obtained from the patients before performing closed reduction and plaster application without using any kind of anesthesia on an OPD basis.

**Figure 2:**
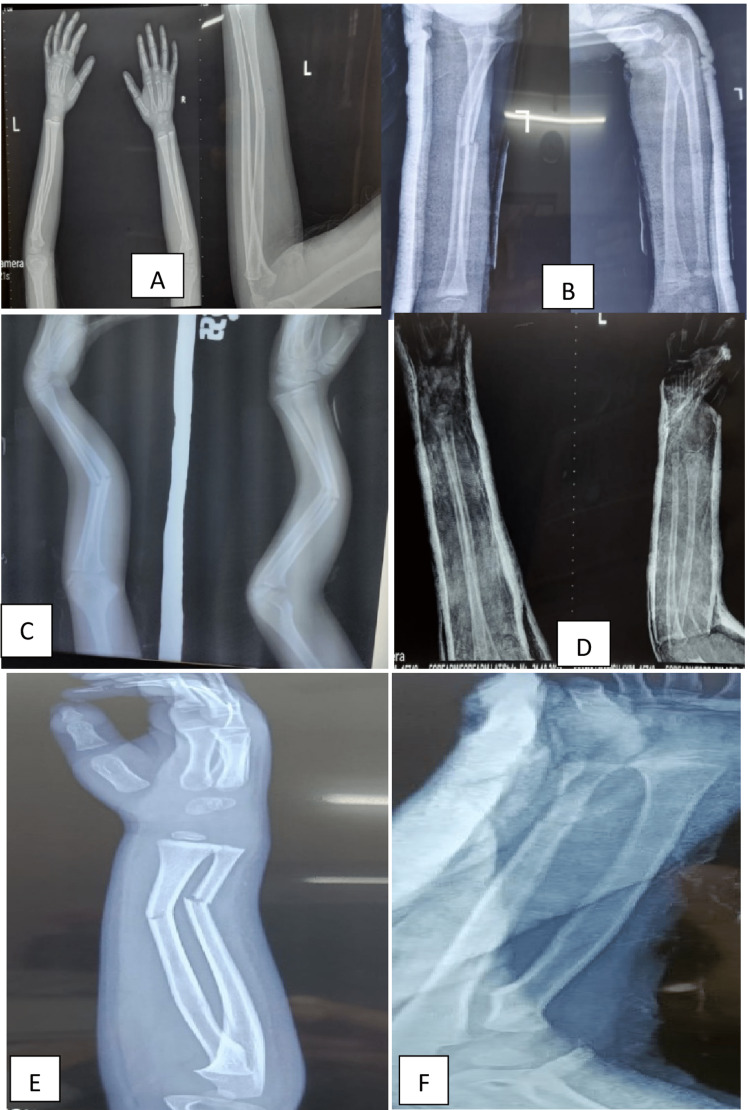
Pre-reduction and post-reduction X-rays of forearm fracture. (A) Pre-reduction X-ray showing greenstick fracture radius ulna. (B) Post-reduction X-ray. (C) Pre-reduction X-ray showing angulated fracture radius ulna. (D) Post-reduction X-ray. (E) Pre-reduction X-ray showing angulated fracture distal radius ulna. (F) Post-reduction X-ray.

**Figure 3:**
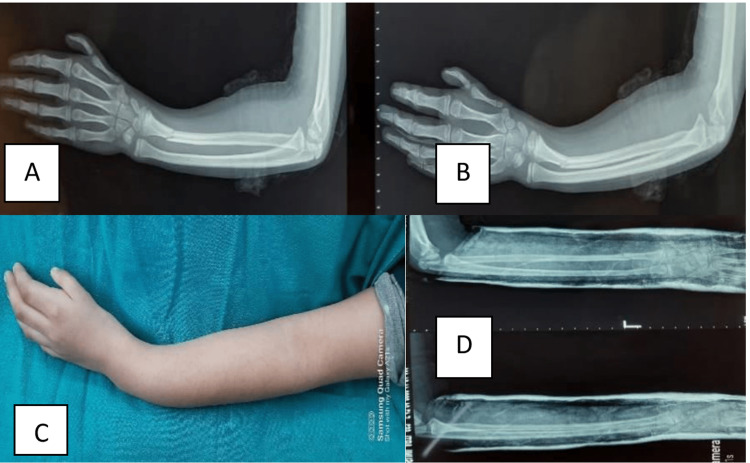
Pre-reduction and post-reduction X-rays of forearm fracture (A, B) Pre-reduction X-ray of forearm showing greenstick fracture distal radius. (C) Clinical picture showing deformity at wrist. (D) Post-reduction X-ray showing satisfactory alignment.

The inclusion criteria were as follows: children between 3 and 16 years of age who had an isolated closed forearm or wrist fracture, with ≥10° of angulation in any plane in the fractured bone. The exclusion criteria were as follows: children with open fractures, fracture dislocation (e.g., Monteggia), neurovascular injuries, polytrauma or multiple injuries, grossly displaced fractures, overriding fractures, or pathological fractures (Figures 2, 3). All closed reductions were performed by a single orthopedic surgeon with the help of an orthopedic-trained operation-room assistant and nurse in an OPD setting without using any anesthesia. 

We allowed the presence of study group parents during closed reduction in the plaster room, so that the child does not get worried, and feels comfortable, relaxed, and cooperative; however, in the control group where anesthesia was used, parents of control groups were not allowed inside the operation theater. They were allowed to watch on CCTV in the waiting hall. 

Reduction technique

After the children were placed in a supine position on a couch, one person would hold the arm, and another person would hold the hand, keeping the elbow flexed at 90°; then, longitudinal traction was applied, and gentle correction of the angulation was done until the clinical alignment was restored. All closed reductions were done under an image intensifier while wearing a lead apron. The children were also protected by lead aprons, covering them up to their knees. No more than two reduction attempts were made.

A below-elbow plaster cast was applied for isolated distal radius fractures through the metaphysis, and an above-elbow plaster cast was applied for mid-shaft radius ulna fractures. The child’s hand was held by the assistant while the surgeon applied the casting material around the arm. All plaster casts were molded opposite to the angulation deformity.

We observed a target cast index of 0.7 so that the cast achieved the contour of the forearm. We did not record whether the plaster in the present study was bivalved. Immediate post-reduction alignment was confirmed using an image intensifier.

Plaster cast care instructions

Pictorial and written instructions for the cast were provided to and discussed with all parents. Special attention was given while educating the children and their parents about the expected pain, which may appear after reduction. All children were advised to keep the arm in an elevated position so that soft tissue swelling and pain could be minimized. Parents were told not to use painkillers as routine but only in the case of unbearable pain.

We did not prescribe any kind of analgesic for any child after closed reduction in both groups. Children of the study group were discharged from the OPD following a short monitoring period (typically 10-15 minutes), during which tolerance of the procedure and pain was monitored. However, children of the control group were discharged from the ward following a short monitoring period (typically 4-6 hours), during which tolerance of the given anesthesia, procedure, and pain was monitored.

We took a post-reduction plain X-ray both immediately after the procedure and then again after seven days to assess the fracture alignment. We changed the plaster cast on the seventh day only if it became loose due to a decrease in soft-tissue swelling. At this time, the patients were asked about any need for pain medications or whether their children faced any complications. Mobile calls and WhatsApp were used to get feedback from the parents about their satisfaction with the treatment.

Sample size calculation

In the present study, we attempted to estimate the sample size based on the difference in mean parents’ satisfaction between both groups. However, there was no literature available concerning parent satisfaction with their child’s fracture care in general practices or hospitals. Therefore, we based our sample size calculation on feasibility. With a 5% two-sided significance level, a statistical power of 80%, and two treatment groups of unequal size, a sample size of 175 participants (113 in the study group and 50 in the control group) was determined to be feasible and sufficient to demonstrate effect sizes of 0.4 (small to medium) or above.

Statistical analysis

We used SPSS software, ver. 19 (SPSS Inc., an IBM Company; Chicago, IL, USA) and MS Excel software for analysis. We performed a power analysis for the sample size calculation; type I error (α) was set at 0.05, the power of the test was 0.90, and the sample size appropriate to test our hypothesis was calculated, with a confidence of 75. Pain intensity was evaluated by a visual analog scale (VAS), where patients were asked to rate the pain they experienced using the VAS. We also performed either a t-test or the Wilcoxon rank-sum test for continuous variables to assess differences in the outcome scores between both treatment groups.

Measurement of parents’ satisfaction in both groups

Primary outcome measure: We used the Patient (in this study, Parents) Satisfaction Questionnaire Short Form (PSQ-18) at 0 and 7 days after treatment [29].

Secondary outcome measures: Parent satisfaction was measured using the PSQ-18, and pain scores and treatment complications, as well as time spent in the hospital (waiting time and treatment time), was recorded, with all assessments done 0 and 7 days after treatment.

During the primary analysis, mean parents’ satisfaction at 0 days and 7 days after closed reduction was compared between the two settings, with differences presented with 95% confidence intervals (CIs). In addition, multivariable regression models were used, with patient satisfaction at 0 days as the dependent variable, and treatment type as well as potential confounders (e.g., age, sex, and type of fracture) as independent variables.

Subsequently, during the secondary analyses, we used multivariable regression models to estimate associations of mean parents’ satisfaction scores with other potential determinants (e.g., pain score, the need for analgesics, complications, and time spent in the hospital).

## Results

In the present study, the 113 children who underwent closed reduction without using any anesthesia with plaster cast application constituted the study group, and the 50 children who underwent closed reduction under some form of anesthesia with plaster cast application constituted the control group.

The average age of children in both groups was 9.5 years (3.5-16.2 years). Eighty-three (73.4%) children in the study group were male, and 36 (72%) in the control group were male. Fifty-seven (50.4%) children were between seven and 10 years of age in the study group, and 28 (56%) were between 10 and 14 years of age in the control group. The right upper limb was involved more than the left, affecting 75 (66.3%) children in the study group and 29 (58%) children in the control group, as shown in Table 1.

**Table 1:** The demographic characteristics of the study and control groups.

Parameters	Study group n=113	Control group n=50	p-Value
Age (in years)			0.623
3-6	31 (27.4%)	13 (26%)	
7-10	57 (50.4%)	09 (18%)	
10-16	25 (22.1%)	28 (56%)	
Sex			0.612
M	83 (73.4%)	36 (72%)	
F	30 (26.5%)	14 (28%)	
Side of injury			0.771
Right	75 (66.3%)	29 (58%)	
Left	38 (33.6%)	21 (42%)	
Time since injury			0.674
>1 hour	58 (51.3%)	24 (48%)	
1-4 hours	34 (30%)	19 (38%)	
4-10 hours	21 (18.5)	7 (14%)	

The most common mechanisms of injury included ground-level falls, injuries while on playgrounds, and injuries during sporting activities. Furthermore, 82 children had radius or ulna fractures, and 31 were isolated distal radius fractures. In addition, 58 (51.3%) children were able to present within less than 1 hour after injury in the study group, compared to 24 (48%) in the control group (p=0.674).

The average angulation in all these fractures was 25° (range: 10°-50°). Satisfactory reduction, as defined by ≤10° of residual angulation, was achieved in 96.8% of children. The average immediate post-reduction angulation was 3° (range: 5°-12°), as measured by the greatest angulation in any plane (Table 2).

**Table 2:** Type of fracture and the degree of angulation in both groups.

Type of fracture	Degree of angulation	Number of children in the study group n=113	Control group n=50
Isolated distal radius	<20	14	7
Isolated distal radius	>20	17	9
Shaft radius ulna both	20-30	39	16
Shaft radius ulna both	30-50	24	10
Shaft radius ulna both	10-20	19	8

Three children in the study group had a residual angulation of 17° post-reduction, which was corrected to 8° after a repeat reduction during the follow-up visit, seven days later. None of the children in the control group had more than 10° of angulation immediately after the reduction.

In the present study, 11 children (12.4%) from the study group were given paracetamol or ibuprofen for pain relief at home after their closed reduction. However, in the control group, 11.4% of children needed analgesics for pain relief at home after their closed reduction. Three children from the control group required antiemetics for nausea and vomiting after general anesthesia. Two children were initially given a regional block that was later converted into general anesthesia because of its failure. We did not record data for any parents who returned for pain associated with the plaster cast or excessive swelling during the first week following the closed reduction and plaster application. 

A minimum time of 1.11±1.08 hours was spent in the hospital by 41 (36.2%) children of the study group, counting from the time of consultation until discharge from the hospital after closed reduction without anesthesia. However, this was 6.13±3.03 hours in 17 (34%) children in the control group (p=0.031). The control group spent more time in the hospital on average because of the formalities of admission, investigations and pre-anesthesia check-up, anesthesia time, procedural time, recovery time, and time taken in the preparation of discharge (Table 3).

**Table 3:** Average time spent in the hospital to get fracture reduction, starting from consultation till discharge (in mean±SD). *Student's t-test. Time spent was calculated from the time of consultation till discharge from hospital (discharge in the case of the study group means when the parents were told to go home after seeing the post-reduction X-ray by the surgeon and in the case of the control group, it was when parents were told to go home after seeing both the post-reduction X-ray by the surgeon and the formal discharge slip).

Time spent in hospital in hours (mean±SD)	Study group	Time spent in hospital in hours (mean±SD)	Control group	p-Value*
1.11±1.08	41 (36.2%)	6.13±3.03	17 (34%)	0.031
2.09±1.79	35 (30.9%)	6.54±2.30	27 (54%)	0.045
3.23±1.13	37 (32.7%)	7.43±2.35	06 (12%)	0.001

Six children (12%) in the control group had spent 7.43±2.35 hours because they presented with a full stomach (eaten meal), and the anesthesiologist had to wait for a few hours before giving general anesthesia.

In the study group, 103 parents (92%) responded to follow-up through 16 weeks on the phone: 56 parents physically came to the hospital and 47 responded on WhatsApp. Three parents physically visited the hospital, and we lost track of seven children who did not report after six weeks post-reduction. However, in the control group, only two children did not report after five weeks post-reduction. All of these parents reported that their child had returned to full function and was pain-free; they felt that their fracture was corrected without much hassle and that the medical treatment was satisfactory. 

In addition to child-related factors such as age, sex, fracture type, and socioeconomic status, the medical aspects of care given by healthcare professionals are the most important determinants of parents’ overall satisfaction.

After closed reduction, the study group children where the reduction was done without anesthesia were happier than the control group children as they were able to reach home much earlier and able to meet their family members. However, it was only subjective and we did not have any objective data. 

A summary of overall scores for the six subscales in both groups is shown in Table 4. Measured on a five-point scale, with 1 meaning low satisfaction and 5 meaning high satisfaction, we observed a significant difference in the mean score for general satisfaction in the study group (4.62±0.93) compared to that in the control group (2.15±0.11) at 0 days post-treatment (p=0.001). We also saw a significant difference in the mean score for time spent in the hospital, with 4.82±0.17 for the study group and 2.37±0.09 for the control group (p=0.002). 

**Table 4:** Parents Satisfaction Questionnaire Short Form scores (PSQ-18) on 0 and 7th days in both groups *Indicates statistically significant. Financial aspect not considered because free treatment was given to all patients in the hospital (transportation cost was also not considered as it was beyond the scope of this study).

Parameters	Study group in mean±SD		Control group in mean±SD		p-Value
	On 0 day	On 7th day	On 0 day	On 7th day	
General satisfaction	4.62±0.93	4.92±0.91	2.15±0.11	2.19±0.31	0.001*
Technical quality	4.32±0.24	4.77±0.66	4.11±0.33	4.17±0.22	0.621
Interpersonal manner	4.67±0.64	4.87±0.71	4.78±0.76	4.72±0.79	0.655
Communication	4.22±0.54	4.70±0.94	4.11±0.55	4.44±0.13	0.874
Time spent in hospital (in hours)	4.82±0.17	4.65±0.38	2.37±0.09	2.67±0.12	0.002*
Accessibility and conveniences	4.50±0.39	4.95±0.62	4.28±0.03	4.53±0.21	0.532
Financial aspect	NA		NA		NA

We did not observe any significant differences in technical quality, interpersonal manner, or communication scores. As far as financial scores were concerned, our hospital is a government-run hospital that provided treatment to all patients free of cost. Transportation cost was not studied, as it was beyond the scope of the present study. The quality of reduction in both groups was equal with satisfactory alignments.

Multivariate regression analysis was done to assess differences in various variables in both groups, with age, sex, and fracture type being insignificant variables. However, the use of analgesics was significant in the control group (risk ratio=2.82, 95% CI=1.43-4.08, p=0.027) as compared with the study group (risk ratio=1.89, 95% CI=1.03-3.05, p=0.052).

We noted a significant difference in time spent in the hospital in the study group (risk ratio=1, 95% CI=1.03-3.10, p=0.001) as compared with the control group (risk ratio=1.13, 95% CI=1.01-4.81, p=0.001). Moreover, we also observed a significant difference in the level of satisfaction among parents in the study group (risk ratio=1, 95% CI=1.03-4.18, p=0.001) as compared with the control group (risk ratio=1, 95% CI=1.06-3.07, p=0.001), as shown in Table 5.

**Table 5:** Multivariate regression analysis of the association between both groups ARR: absolute risk reduction; CI: confidence interval.

Variables	Study group		p-Value	Control group		p-Value
	ARR	95% CI		ARR	95% CI	
Age						
3-10 years	1.00	1.03-1.02	0.612	1.06	1.02-1.01	0.632
10-16 years	1.09	1.00-1.01		1.07	1.00-1.01	
Sex						
M	1.40	1.10-1.09	0.654	1.30	1.09-1.04	0.677
F	1.03	1.05-1.02		1.06	1.01-1.03	
Types of fractures						
Distal radius	1.04	1.01-1.00	0.751	1.02	1.06-1.09	0.784
Both bone	1.02	1.08-1.04		1.05	1.07-1.00	
Use of analgesics post reduction						
Yes	1.89	1.03-3.05	0.052	2.82	1.43-4.08	0.027
No	0.01	1.00-0.70		0.07	1.09-2.90	
Time spent in hospital						
1-4 hours	1.00	1.03-3.10	0.001	1.13	1.01-4.81	0.001
4-8 hours	1.09	1.00-3.09		1.09	2.03-5.781	
>8 hours	1.03	1.12-3.11		1.10	2.00-7.220	
Parents' satisfaction						
Highly satisfied	1.00	1.03-4.18	0.001	1.00	1.06-3.07	0.541
Satisfied	1.09	1.00-2.23		1.11	1.00-1.88	

## Discussion

Although closed reduction and improvement of fracture alignment without anesthesia is not a novel approach, we sometimes do it for mildly angulated fractures. Upon reviewing the literature, we did not find many studies on closed reduction without using any kind of anesthesia in pediatric fractures as a routine standalone method. Many authors have instead focused on comparing the outcomes of closed reduction using different kinds of anesthesia, including hematoma blocks, Bier blocks, peripheral nerve blocks, procedural sedation, and general anesthesia [13-16,19]. Anesthesia of any kind is not free of side effects or complications, which can be local, regional, or general. These complications can include nausea/vomiting, aspiration pneumonitis, arrhythmia, anaphylaxis, hypotension, malignant hyperthermia, seizure, respiratory depression, and even death [21-24].

A recent study by Livingstone et al., who studied 54 children, reported good outcomes and satisfaction among parents after closed reduction without the use of any kind of anesthesia [30]. With more recent literature on the risks of both local and systemic anesthetics and analgesics in children, it may become acceptable to treat greenstick angulated forearm and distal-end radius fractures in children without the use of any kind of anesthesia [31-33]. Given the aforementioned complications associated with anesthetics and anesthesia, we started our study and tried to evaluate the effects of closed reduction without the use of any anesthesia, and measured the level of satisfaction among parents.

Limitations and strengths of the present study

Our study had a few limitations. First, the results may not be generalizable and may be impacted by the treating surgeon’s experience, skill, and confidence in performing anesthesia-free closed reductions. Second, although we were unable to obtain formal financial data related to anesthesia, facility fees suggest that costs may be lowered by using our approach.

The major strength of the present study was the 100% follow-up rate until five weeks in both groups, which allowed all parents to be asked about the use of analgesics at home. Furthermore, a thorough discussion was held with the child and their parents about post-reduction pain expectations and the need to minimize soft-tissue swelling.

No analgesics were prescribed to children in the study group after closed reduction, and only the parents of 11 children (12.4%) reported that they felt their child needed painkillers. The low rate of analgesics usage in the present study suggests that post-reduction pain was adequately controlled with the non-pharmacological methods used, and the parents were likely satisfied with their child’s pain management.

As far as the parent and child satisfaction is concerned, the use of analgesics is not a validated substitute for the larger outcome. The parents of 11 children (12.4%) from the study group reported that their child experienced more pain at night and that they had to give painkillers only at night for two days at most. This result may be because these 11 children were more anxious and had less pain tolerance following closed reduction; furthermore, it may also indicate that this method of anesthesia-free closed reduction may not be suitable for all children. 

## Conclusions

Although the results of the present study showed that some children required analgesics, there was a decrease in time spent in the hospital post-reduction without anesthesia, with high parent satisfaction. However, this approach may not be suitable for children with some form of anxiety, or those with psychiatric or mental disabilities, who may require some form of anesthesia. Moreover, closed reduction without anesthesia should only be done by an orthopedic surgeon with great caution and by gentle manipulation.

Our method is an effective alternative option for children who present with greenstick and angulated fracture of the forearm bone and wrist fracture (fracture distal-end radius), and who require gentle manipulation to achieve satisfactory alignment. The method also avoids all complications associated with anesthesia, whether local, regional, or general. Given the above results, we strongly believe that additional prospective comparative studies of this method are required to create definitive guidelines about anesthesia-free closed reduction.
